# Effectiveness and safety of echinocandins combination therapy with the standard of care compared to the standard of care monotherapy for the treatment of invasive aspergillosis infection: a meta-analysis

**DOI:** 10.3389/fphar.2024.1500529

**Published:** 2024-12-19

**Authors:** Yazed Saleh Alsowaida, Bader Alshoumr, Shuroug A. Alowais, Khalid Bin Saleh, Alia Alshammari, Kareemah Alshurtan, Haytham A. Wali

**Affiliations:** ^1^ Department of Clinical Pharmacy, College of Pharmacy, University of Hail, Hail, Saudi Arabia; ^2^ Department of Health Informatics, College of Public Health, University of Hail, Hail, Saudi Arabia; ^3^ Department of Pharmacy Practice, Pharmacy College, King Abdulaziz University for Health Sciences, King Abdullah International Medical Research Center, Pharmaceutical Care Department, King Abdulaziz Medical City, Riyadh, Saudi Arabia; ^4^ Department of Pharmaceutics, College of Pharmacy, University of Hail, Hail, Saudi Arabia; ^5^ Department of Internal Medicine and Adult Critical Care, College of Medicine, University of Ha’il, Hail, Saudi Arabia; ^6^ Department of Pharmacy Practice, College of Clinical Pharmacy, King Faisal University, Al-Ahsa, Saudi Arabia

**Keywords:** aspergillosis, echinocandins, meta-analysis, anidulafungin, caspofungin

## Abstract

**Background:**

This meta-analysis aims to evaluate the effectiveness and safety of combining echinocandins with standard of care (SOC) antifungal drugs for treating invasive aspergillosis infection (IAI).

**Method:**

We searched PubMed, Embase, and Cochrane Library from their inception to 25 July 2024. Our outcomes included clinical cure, mortality, and adverse drug reactions (ADRs). We compared echinocandins in combination with SOC antifungal agents against SOC monotherapy therapy. We used the random-effects model for the meta-analysis, and our estimated effects were reported as odds ratios (ORs) with 95% confidence intervals (CI).

**Results:**

Ten studies were included in our meta-analysis comprising 1100 patients: 415 were in the echinocandin combination groups, and 685 were in the SOC groups. The clinical cure rate (OR 1.35, 95% CI: 0.75–2.42, *p* = 0.27), mortality (OR 0.90, 95% CI: 0.50–1.63, *p* = 0.73), and ADRs rate (OR 0.95, 95% CI: 0.49–1.82, *p* = 0.87) were not statistically different in echinocandins combination with SOC compared to SOC monotherapy. Notably, there is a signal for a better clinical cure rate in echinocandins in combination with SOC.

**Conclusion:**

Our meta-analysis found no differences in clinical cure and mortality rate when using combination therapy of echinocandin antifungal agents with the SOC compared to SOC monotherapy. However, there is a signal for better outcomes with the echinocandins combination group. The ADRs in the echinocandins combination group were not worse than SOC monotherapy.

## 1 Introduction

Invasive aspergillosis is a mycotic infection caused by the ubiquitous mold in the environment–*Aspergillus fumigatus,* and causes infections in the lungs (most commonly), skin, central nervous system, and sinuses (Chabi et al., 2015; Lamoth and Calandra, 2022). The cases of invasive aspergillosis infection (IAI) are rising globally (Thompson and Young, 2021). The estimated annual global incidence of IAI from 120 countries (Africa, Asia and Pacific, Latin America and the Caribbean, Eastern Europe and Central Asia, Western Europe and North America, Middle East and North Africa) was 2 116 362 cases, with a crude mortality rate of 72% (Denning, 2024). Aspergillosis is associated with a wide range of clinical syndromes, such as chronic pulmonary aspergillosis, sinus disease, infection of the central nervous system, and others (Thompson and Young, 2021). Factors attributable to the increasing IAIs include growing immunosuppressed populations, intensive care unit admission, and severe respiratory viral infections like influenza and SARS-CoV-2 infections. Notably, IAI is challenging to diagnose because it does not have unique clinical manifestations and it takes a long time to detect (Wang et al., 2022).

Antifungal pharmacotherapy is the standard of care (SOC) for treating IAI (Thompson and Young, 2021). Some patients with IAI may need surgery in addition to antifungal therapy (Patterson et al., 2016). The current Infectious Diseases Society of America (IDSA) guideline for IAI recommends antifungal treatment with triazole antifungal agents, preferably voriconazole. Amphotericin B, with its varying formulations, is appropriate when voriconazole cannot be used or indicated as salvage therapy. Echinocandin antifungal agents are not recommended as a monotherapy for treating IAI. The IDSA guideline recommends echinocandins for IAI as salvage therapy in combination with the standard of care (Amphotericin B or triazole antifungal agents).

Preclinical studies revealed that combining azole and polyene antifungal agents yields synergistic or additive effects (Patterson et al., 2016; Petraitis et al., 2017). Yet, evidence is conflicting regarding the effectiveness of preclinical data. An experimental study by Petraitis et al. evaluated a combination therapy of isavuconazole with micafungin for treating invasive pulmonary aspergillosis in an animal model. The authors found that the isavuconazole/micafungin combination therapy yielded a dose-dependent decrease in fungal burden, pulmonary injury, and prolonged animal survival (Petraitis et al., 2017). Furthermore, another study by Jeans et al. evaluated the combination of voriconazole and anidulafungin against triazole-resistant Aspergillus fumigatus in an *in-vitro* invasive pulmonary aspergillosis (Jeans et al., 2012). The authors concluded that the combination therapy of voriconazole and anidulafungin had an apparent additive effect and reduced galactomannan concentration in the endothelial compartment.

Evidence for the effectiveness and safety of echinocandin combination therapy with the SOC against IAI is conflicting and inconclusive. A study by Singh et al. evaluated a combination of voriconazole and caspofungin for IAI and found that combination therapy had preferable outcomes to monotherapy (Singh et al., 2006). On the other hand, a study by Raad et al. evaluated the voriconazole and caspofungin combination for IAI and concluded that it did not result in improved outcomes compared to monotherapy (Raad et al., 2015). Furthermore, published meta-analyses have limitations, such as not including all the published studies and combining animal and human studies, which pose remarkable heterogeneity in the results estimates (Panackal et al., 2014; Zhang et al., 2014). Therefore, robust and updated evidence from a well-designed meta-analysis is warranted. Therefore, this study aims to evaluate the effectiveness and safety of combining echinocandin with the SOC for treating IAI quantitatively, employing meta-analysis to update the available evidence.

## 2 Materials and methods

We followed the Preferred Reporting Items for Systematic Reviews and Meta-Analyses (PRISMA) and the Meta-Analyses of Observational Studies in Epidemiology (MOOSE) guidelines during our meta-analysis (Page et al., 2021; Stroup et al., 2000). The checklists for the PRISMA and MOOSE guidelines are available in the Supplementary Material.

### 2.1 Literature source

We systematically searched PubMed, Cochrane Library, and Embase databases from inception until 25 July 2024. The search was executed by cross-searching keywords with Medical Subject Headings using the following concepts: aspergillosis, echinocandin antifungal drugs, and all other systemic antifungal drugs. The complete search strategy is available in Supplementary Material.

### 2.2 PICOS criteria and study selection

We used the following adjusted Population, Intervention, Comparator, Outcomes, and Studies (PICOS) criteria (Methley et al., 2014): 1) Population: adult patients 18 years and older with IAI; 2) Intervention: aspergillosis-active agents (amphotericin, voriconazole, posaconazole, itraconazole, or isavuconazonium) combined with an echinocandin (micafungin, caspofungin, anidulafungin, or rezafungin); 3) Comparator: the SOC monotherapy for IAI (amphotericin, amphotericin, voriconazole, posaconazole, itraconazole, or isavuconazonium); 4) Outcomes: clinical cure, mortality, microbiological cure, and safety; Studies: randomized controlled trials (RCT) and observational studies. Investigators (YSA and HY) reviewed the titles and abstracts of identified studies and evaluated their eligibility based on the pre-determined PICOS criteria. We excluded editorials, commentaries, case reports, case series, pharmacokinetic (PK) studies, pharmacodynamic (PD) studies, animal studies, and publications in non-English language. The following keywords were used in our search: aspergillosis, invasive aspergillosis, pulmonary aspergillosis, echinocandins, anidulafungin, caspofungin, micafungin, rezafungin, isavuconazole, itraconazole, voriconazole, and amphotericin.

### 2.3 Data extraction

Two investigators (YSA and HW) separately screened identified studies and performed a full-text review of potentially relevant studies for eligibility. Discordance was resolved with discussion and consensus, and unsettled matters were evaluated by a third investigator (KBS). For included studies, we extracted the first author’s name and publication year, the location of the study population, the sample size of each arm, and the regimen used in each study.

### 2.4 Quality assessment

We used the Newcastle-Ottawa Scale (NOS) to assess the quality of the included observational studies (Wells et al., 2024) Based on their scores, studies were classified into low quality (1–3 stars), medium quality (4–6 stars), and high quality (7–9 stars). For the quality assessment of the RCTs, we used the Jadad score (Jadad et al., 1996) Studies with scores of ≥3 were categorized as high quality, and studies with scores of ≤2 were categorized as low quality.

### 2.5 Summary measures and statistical analysis

The combined measured effects were reported as odds ratios (OR) with a 95% confidence interval (CI). Due to the heterogeneity of the study populations, we used the restricted maximum likelihood random-effects model for the meta-analyses (Borenstein et al., 2010). Additionally, we utilized Cochrane’s Q to calculate the heterogeneity by the weighted sum of squares and the I^2^ statistic to express the percentage of variation due to heterogeneity (Higgins et al., 2003). Statistically significant heterogeneity was defined as a threshold of *p*-value <0.05 for Cochrane’s Q statistics and >30% for I^2^ statistics. An estimation of publication bias was assessed by funnel plots of standard error against the estimated effect. Lastly, the asymmetry of the funnel plot was examined using the Egger linear regression test (Egger et al., 1997).

### 2.6 Subgroup analysis

To further evaluate mortality and clinical cure, we perform sub-group meta-analyses based on the echinocandin regimens used in the studies, treatment intent: primary vs. salvage therapy, and patient populations (hematologic malignancies). Specifically, we computed the overall clinical cure, clinical cure for patients on combination therapy as primary therapy, clinical cure for patients on combination therapy as salvage therapy, clinical cure for caspofungin-based regimens, and clinical cure for patients with hematologic malignancies. Furthermore, for mortality, we performed overall mortality, mortality for patients on combination therapy as primary therapy, mortality for patients on combination therapy as salvage therapy, mortality for caspofungin-based regimens, and mortality for patients with hematologic malignancies.

## 3 Results

### 3.1 Study selection

**FIGURE 1:**
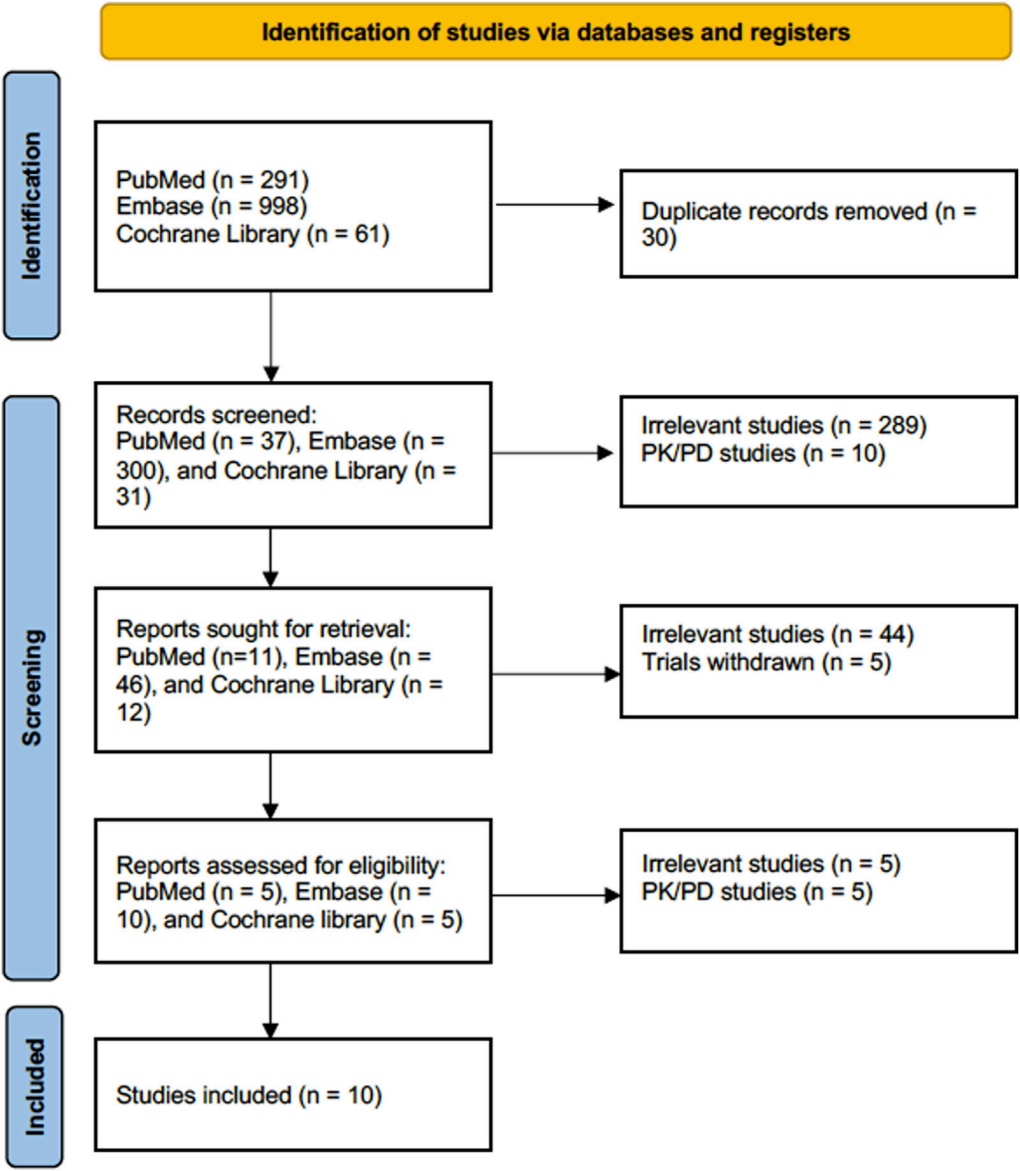
Flow diagram for Preferred Reporting Items for Systematic Reviews and Meta-Analyses (PRISMA).

The literature search strategy resulted in 998 articles in Embase, 291 articles in PubMed, and 61 articles in the Cochrane Library. Notably, some articles were identified from known articles to the investigators. A total of 368 articles were screened after removing duplicate articles. After that, we eliminated 289 articles for being irrelevant and ten articles for being PK/PD studies, leaving us with 69 articles for review. After 49 irrelevant articles were removed, 20 articles underwent a full-text assessment for eligibility. After the full-text evaluation of the articles, nine were removed, and ten were included in the meta-analysis, Figure 1.

Notably, studies by Raad et al. (2015), Mihu et al. (2010), Racil et al. (2013) reported outcomes for echinocandins monotherapy; however, we excluded these outcomes from our analysis for clinical cure and mortality because echinocandins monotherapy is not recommended for treating IAI based on the IDSA guideline (Patterson et al., 2016). Moreover, we included the study by Mihu et al. (2010) in our subgroup analysis for caspofungin-based regimens for clinical cure and mortality because 90% of the patients received caspofungin.

### 3.2 Characteristics of the included studies

Ten studies (Caillot et al., 2007; John and Brizendine, 2016; Marr et al., 2004; Marr et al., 2015; Mihu et al., 2010; Pagano et al., 2010; Raad et al., 2008; Raad et al., 2015; Racil et al., 2013; Singh et al., 2006) were included in the meta-analysis: six retrospective cohort studies (John and Brizendine, 2016; Marr et al., 2004; Mihu et al., 2010; Raad et al., 2008; Raad et al., 2015; Racil et al., 2013), two prospective observational studies (Pagano et al., 2010; Singh et al., 2006), and two randomized trials (Caillot et al., 2007; Marr et al., 2015). All included studies were high quality, except the study by Caillot et al. (2007) was low quality (Caillot et al., 2007; Marr et al., 2015). Six of the included studies were conducted in the United States, three were conducted in Europe, and 1 was a multinational study. The patient populations of the included studies were hematologic malignancies or transplantation. The total number of patients included in the meta-analysis was 1100: 415 patients were in the echinocandin combination groups, and 685 were in the SOC groups. Complete characteristics of the included studies are available in Table 1.

**TABLE 1:** Characteristics of the included studies.

Study	Study design	Location and period of the study	Patient population	Treatment intent	Regimen for echinocandins combination group	Regimen for SOC monotherapy group	Quality assessment score	Quality ranking
Singh et al. (2006)	Multicenter, prospective, observational study	United States from 2003–2005	Organ transplant recipient	Primary therapy for IAI	Voriconazole 6 mg/kg q12 h for 1 day, then 4 mg/kg q12 h + caspofungin 70 mg IV for 1 day, then 50 mg q24 h	Lipid formulation of amphotericin B deoxycholate 5–7.4 mg/kg/day	8 stars	High quality
Raad et al. (2015)	Retrospective cohort study	United States from July 1998 to December 2010	Hematologic malignancy patients with proven or probable IAI	Primary and salvage therapy for IAI	Voriconazole 6 mg/kg q12 h on the first day, then 4 mg/kg q12 h + caspofungin 70 mg IV on day 1, then 50 mg IV q24 h	Voriconazole 6 mg/kg q12 h on the first day, then 4 mg/kg q12 h, or caspofungin 70 mg IV on day 1, then 50 mg IV q24 h	9 stars	High quality
Raad et al. (2008)	Retrospective cohort study	United States from 1999 to 2005	Hematologic malignancy patients with proven or probable IAI	Salvage therapy for IAI	High-dose lipid formulation of amphotericin B ≥ 7.5 mg/kg/day + caspofungin 70 mg IV on day 1, then 50–100 mg q24 h	High-dose lipid formulation of amphotericin B ≥ 7.5 mg/kg/day	9 stars	High quality
Caillot et al. (2007)	Multicenter, pilot, prospective, randomized open trial	France from April 2004 to July 2005	Immunocompromised patients with proven or probable IAI	Primary therapy	Liposomal amphotericin B 3 mg/kg/day + caspofungin 70 mg IV on day1, then 50 mg IV q24 h	High-dose liposomal amphotericin B 10 mg/kg/day	1 star	Low quality
Pagano et al. (2010)	Prospective observational study	Italy from January 2004 to December 2007	Acute myeloid leukemia patients with proven or probable IAI	Primary therapy	Liposomal amphotericin B+ caspofungin, or voriconazole + caspofungin	Amphotericin B, voriconazole, posaconazole, or caspofungin	9 stars	High quality
Mihu et al. (2010)	A retrospective cohort study	United States from August 1993 until June 2008	Hematologic malignancy patients with proven or probable IAI	Salvage therapy for IAI	Liposomal amphotericin B+ caspofungin or anidulafungin	Liposomal amphotericin B	9 stars	High quality
John and Brizendine (2016)	A retrospective cohort study	United States from 2008 to 2015	Hematopoietic cell transplantation patients with galactomannan antigen positivity in serum or bronchoalveolar lavage fluid	Primary therapy	Voriconazole + micafungin, or posaconazole + micafungin	Not reported	7 stars	High quality
Racil et al. (2013)	Retrospective cohort study	Czech and Slovak republics from 1 January 2005 to 31 December 2009	Hematologic malignancy patients with proven or probable IAI	Primary and salvage therapy	Voriconazole + an echinocandin	Voriconazole or amphotericin B	7 stars R	High quality
Marr et al. (2015)	A randomized, controlled, trial	Multinational from 9 July 2008 and 12 May 2011	Hematologic malignancies and hematopoietic cell transplantation patients with possible, probable, or proven IAI	Primary therapy	Voriconazole 6 mg/kg q12 h IV on day 1, then 4 mg/kg q12 h IV for a week, voriconazole 300 mg q12 h PO for 6 weeks + anidulafungin 200 mg IV on day 1, then 100 mg q24 h for up to 4 weeks*	Voriconazole 6 mg/kg q12 h IV on day 1, then 4 mg/kg q12 h IV for a week, voriconazole 300 mg q12 h PO for 6 weeks monotherapy	4 stars	High quality
Marr et al. (2004)	Retrospective cohort study	United States from 1997 to 2001	Hematologic malignancies and hematopoietic cell transplantation patients with proven or probable IAI	Salvage therapy	Amphotericin B 1 mg/kg q24 h or voriconazole 6 mg/kg on day 1, then 4 mg/kg q12 h + caspofungin 70 mg IV on day 1, then 50 mg q24 h	Voriconazole 6 mg/kg on day 1, then 4 mg/kg q12 h	8	High quality

Abbreviation: IAI: invasive aspergillosis infection, *: voriconazole dose can be adjusted based on drug concentration, clinical response, and adverse effects.

### 3.3 Meta-analysis

#### 3.3.1 Clinical cure rate

**FIGURE 2:**
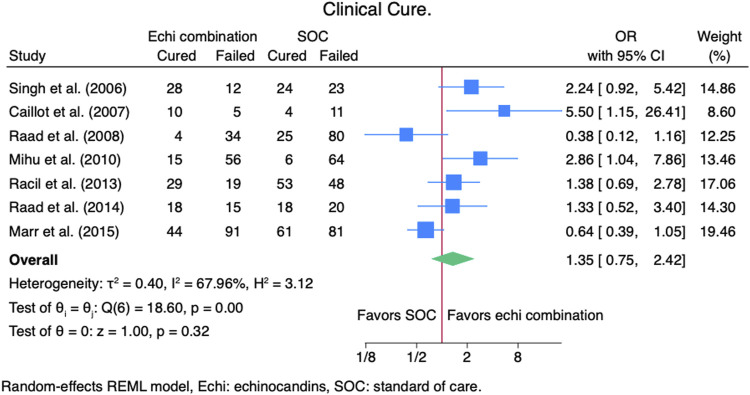
Meta-analysis of clinical cure.

**FIGURE 3:**
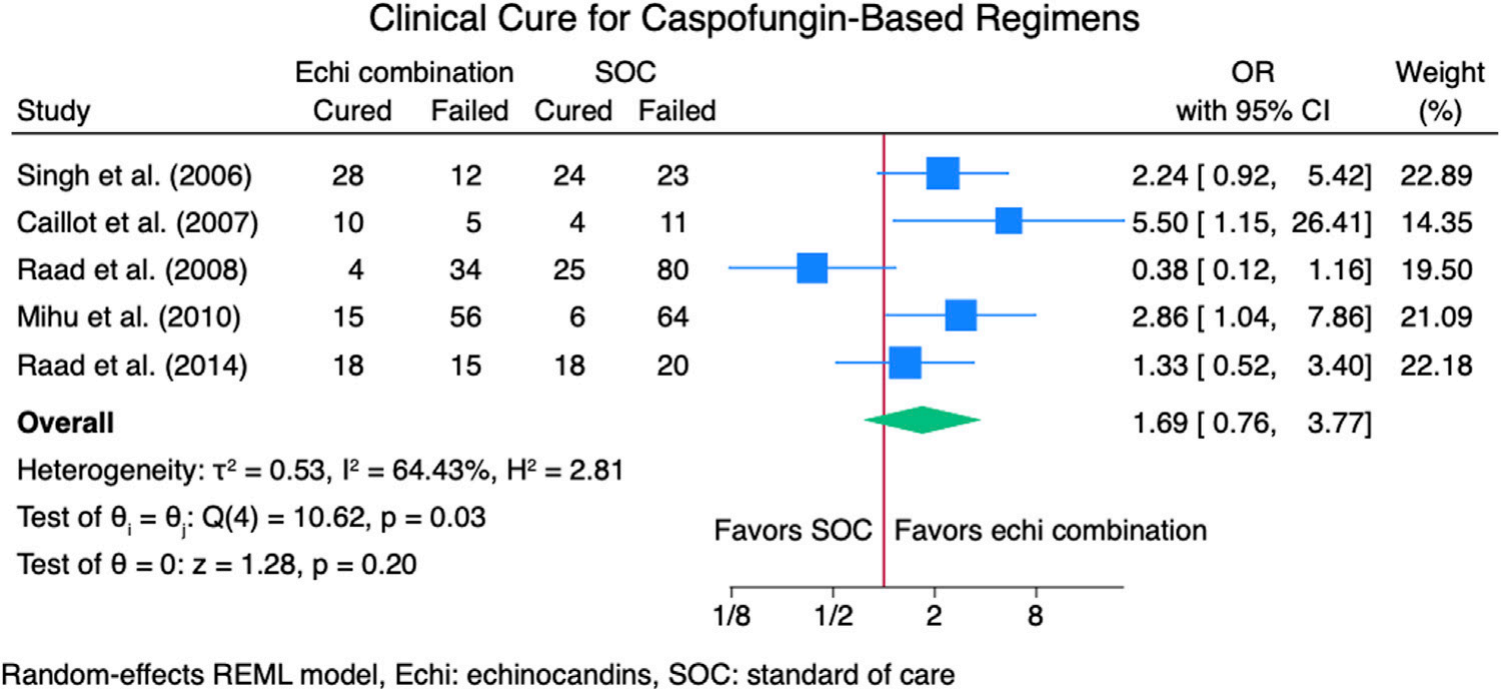
Subgroup meta-analysis for clinical cure rate for caspofungin-based regimens.

**FIGURE 4:**
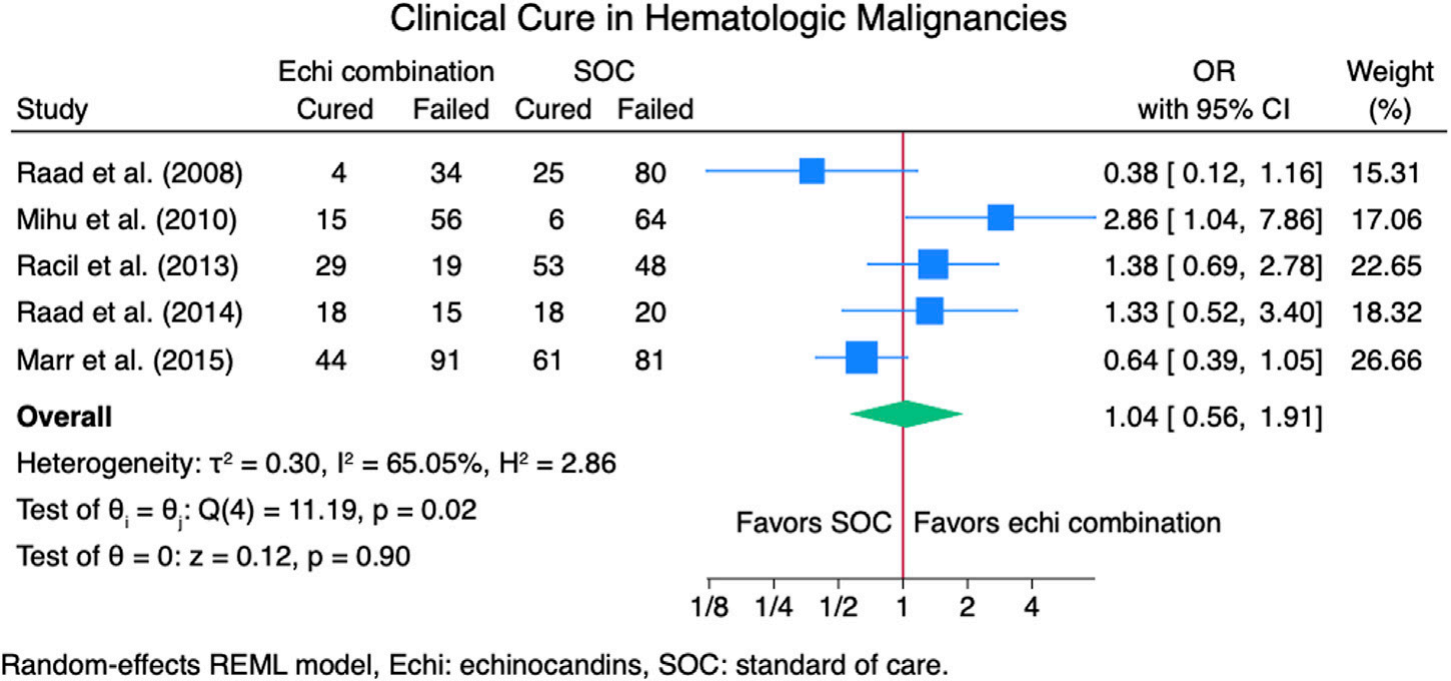
Subgroup meta-analysis for clinical cure rate for patients with hematologic malignancies.

Seven studies (Caillot et al., 2007; Marr et al., 2015; Mihu et al., 2010; Raad et al., 2008; Raad et al., 2015; Racil et al., 2013; Singh et al., 2006) reported clinical cure outcomes for 913 patients: 380 patients in the echinocandin combination therapy group and 533 in the SOC group. The pooled clinical cure rate when using an echinocandin combination therapy was not statistically different from SOC monotherapy: OR 1.35 (95% CI: 0.75–2.42, *p* = 0.27; Figure 2). In the subgroup meta-analysis, when using echinocandins combination therapy for IAI as primary therapy (Caillot et al., 2007; Marr et al., 2015; Raad et al., 2015; Racil et al., 2013; Singh et al., 2006), the clinical cure rate was also not statistically different for echinocandins combination therapy compared to SOC monotherapy; OR 1.37 (95% CI: 0.74–2.53, *p* = 0.31; Supplementary Figure S1). The lack of clinically significant difference in clinical cure rate also persists when using echinocandins combination therapy as a salvage therapy (Mihu et al., 2010; Raad et al., 2008; Raad et al., 2015; Racil et al., 2013) for IAI; OR 1.11 (95% CI: 0.36–3.41, *p* = 0.86; Supplementary Figure S2). Lastly, the clinical cure rate was not statistically different when using combination echinocandin therapy for the subgroup meta-analysis of caspofungin-based combination regiments (Caillot et al., 2007; Mihu et al., 2010; Raad et al., 2008; Raad et al., 2015; Singh et al., 2006); OR 1.69 (95% CI: 0.76–3.77, *p* = 0.20; Figure 3). Notably, there was a signal for better outcomes with echinocandin combination therapies for the overall clinical cure, subgroup analysis for primary IAI therapy, and subgroup analysis for caspofungin-based regimens. Finally, we performed a subgroup meta-analysis for the clinical cure rate for studies comprising hematologic malignancy patients (Marr et al., 2015; Mihu et al., 2010; Raad et al., 2008; Raad et al., 2015; Racil et al., 2013), and the clinical cure rate was similar for the echinocandins combination group compared to SOC monotherapy: OR 1.04 (95% CI: 0.56–1.91, *p* = 0.90, Figure 4).

#### 3.3.2 Mortality rate

**FIGURE 5:**
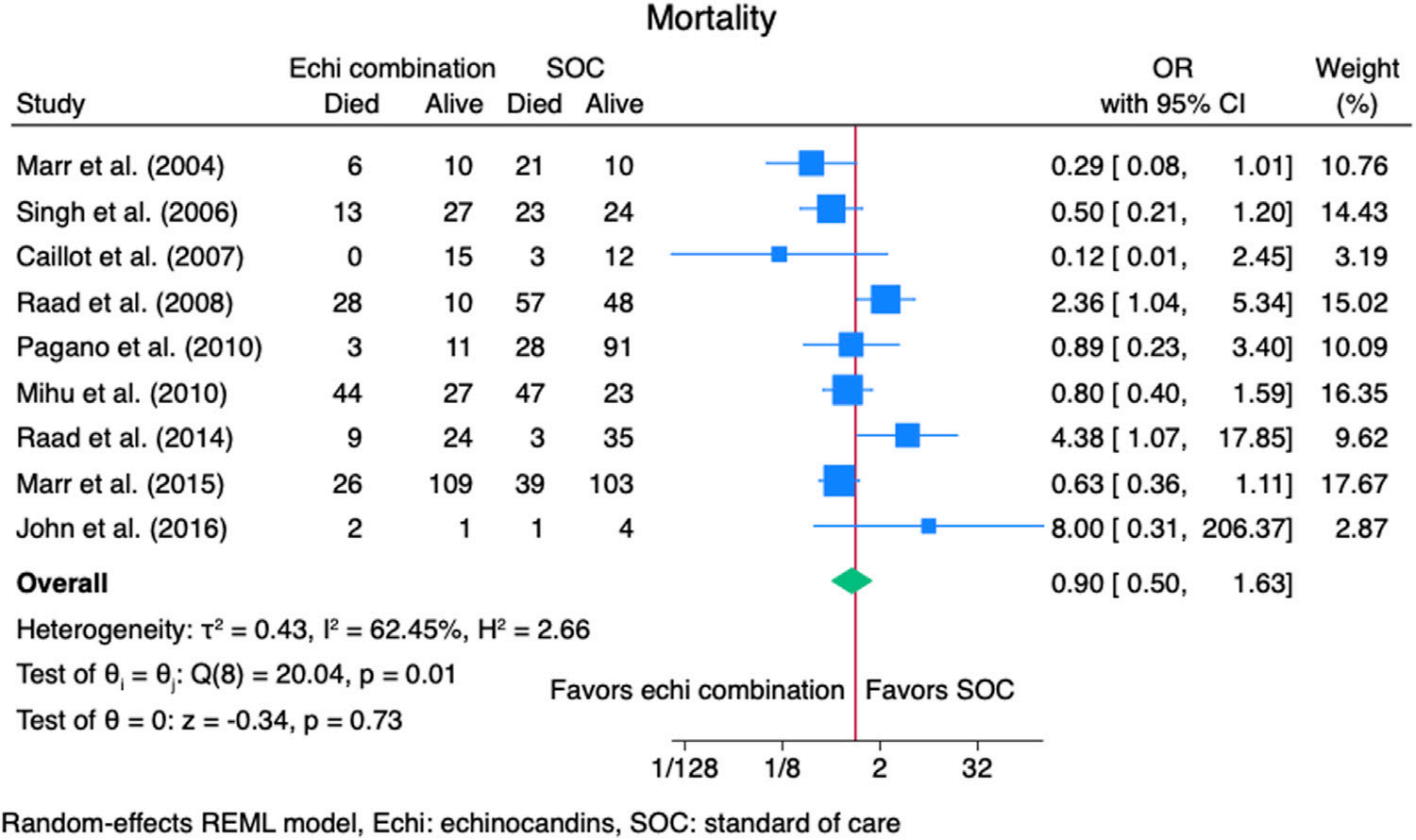
Meta-analysis of mortality rate.

**FIGURE 6:**
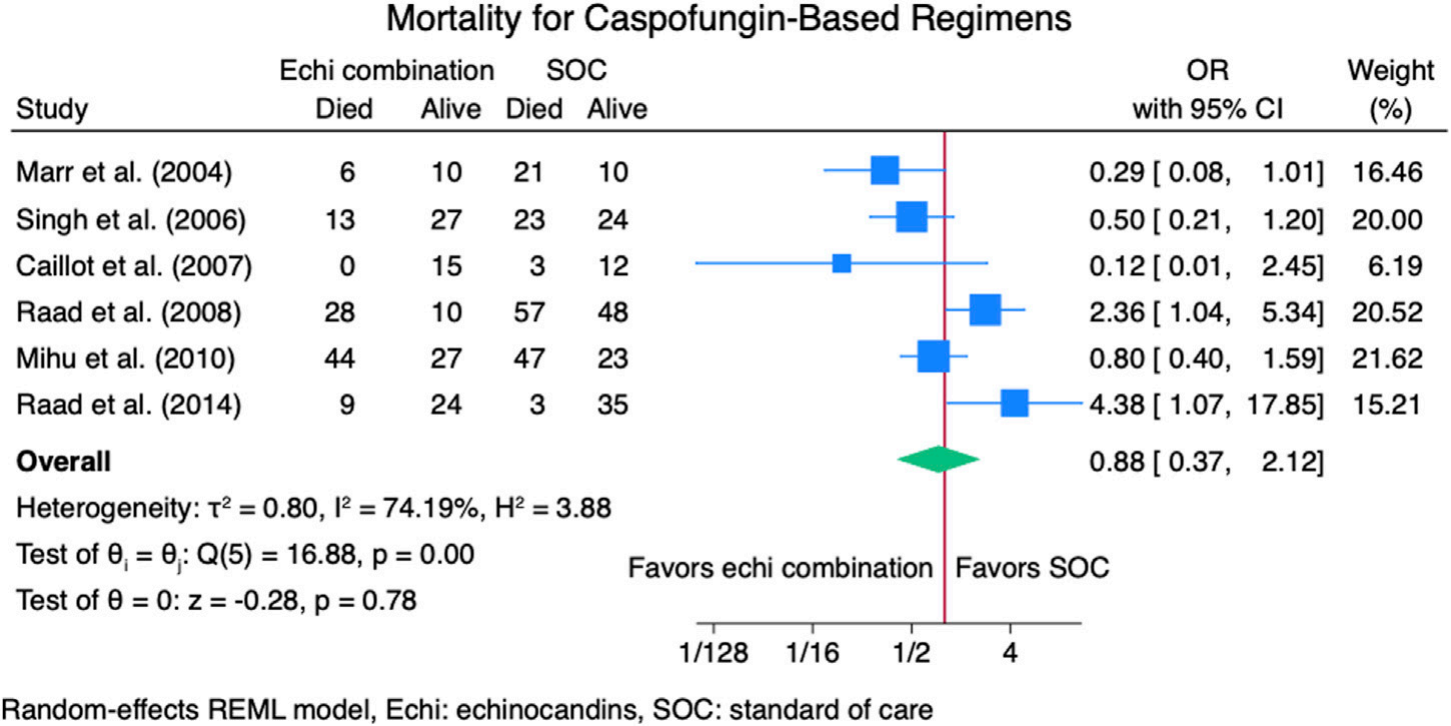
Subgroup meta-analysis of mortality rate for caspofungin-based regimens.

**FIGURE 7:**
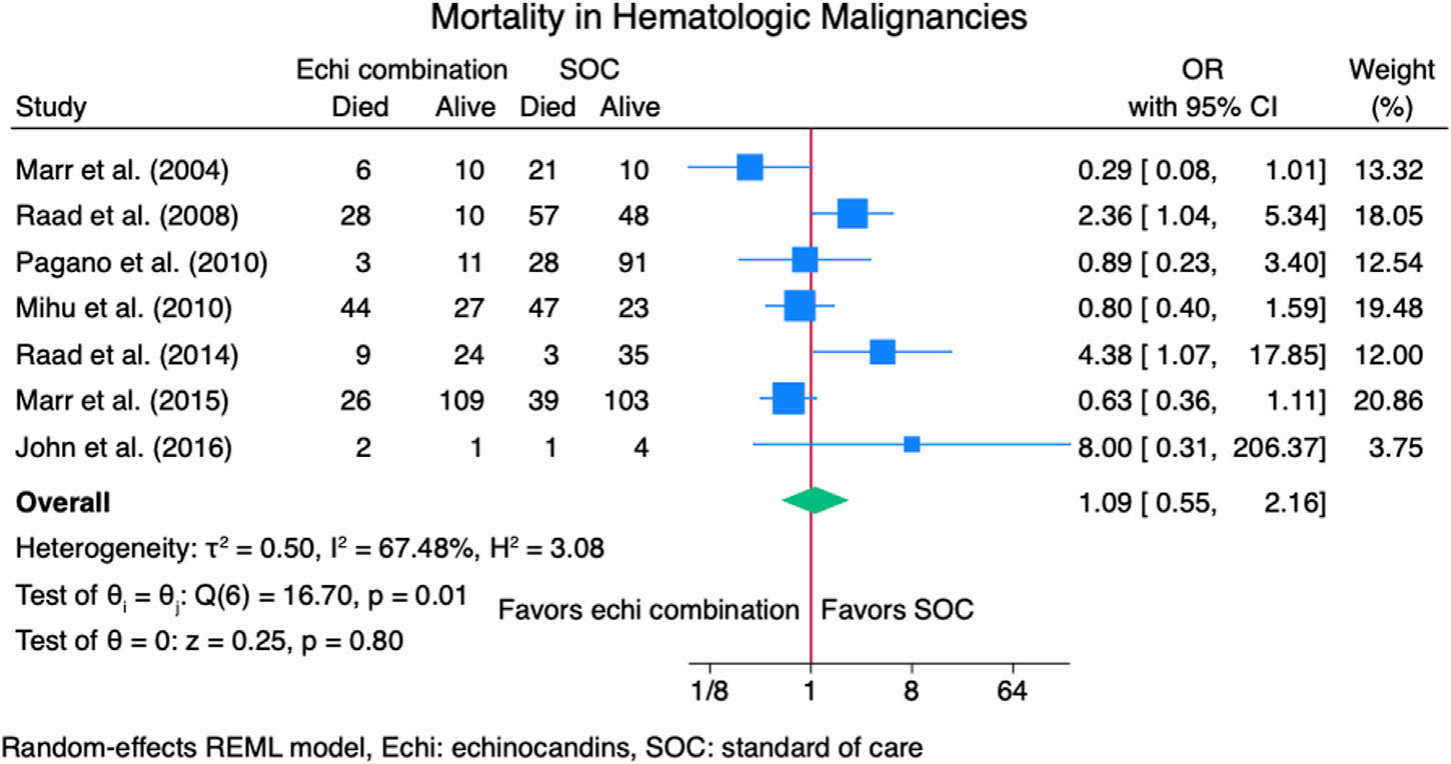
Subgroup meta-analysis of mortality rate for patients with hematologic malignancies.

Nine studies (Caillot et al., 2007; John and Brizendine, 2016; Marr et al., 2004; Marr et al., 2015; Mihu et al., 2010; Pagano et al., 2010; Raad et al., 2008; Raad et al., 2015; Singh et al., 2006) reported mortality outcomes for 937 patients: 365 patients in the echinocandins combination group and 572 in the SOC monotherapy group. The pooled OR when using echinocandins combination therapy was not statistically different from that of SOC monotherapy: OR 0.90 (95% CI: 0.50–1.63, *p* = 0.73, Figure 5). In the subgroup meta-analysis, when using echinocandins combination therapy for IAI as primary therapy (Caillot et al., 2007; John and Brizendine, 2016; Marr et al., 2015; Pagano et al., 2010; Raad et al., 2015; Singh et al., 2006), the mortality rate was not statistically different compared to SOC monotherapy: OR 0.91 (95% CI: 0.41–2.01, *p* = 0.81, Supplementary Figure S3). Furthermore, in the subgroup meta-analysis for salvage therapy (Marr et al., 2004; Mihu et al., 2010; Raad et al., 2008) for IAI, the mortality when using echinocandins combination therapy was not statistically different compared to SOC monotherapy: OR 0.88 (95% CI: 0.28–2.71, *p* = 0.82, Supplementary Figure S4). After all, in the subgroup meta-analysis for caspofungin-based regimens (Caillot et al., 2007; Marr et al., 2004; Mihu et al., 2010; Raad et al., 2008; Raad et al., 2015; Singh et al., 2006), the mortality rate with echinocandins combination therapy was not statistically different compared to SOC monotherapy: OR 0.88 (95% CI: 0.37–2.12, *p* = 0.78, Figure 6). Finally, we performed a subgroup meta-analysis for the mortality rate for studies comprising hematologic malignancy patients(John and Brizendine, 2016; Marr et al., 2004; Marr et al., 2015; Mihu et al., 2010; Pagano et al., 2010; Raad et al., 2008; Raad et al., 2015), and the mortality rate was similar for the echinocandins combination group compared to SOC monotherapy: OR 1.09 (95% CI: 0.55–2.16, *p* = 0.80, Figure 7).

#### 3.3.3 Adverse drug reactions

**FIGURE 8:**
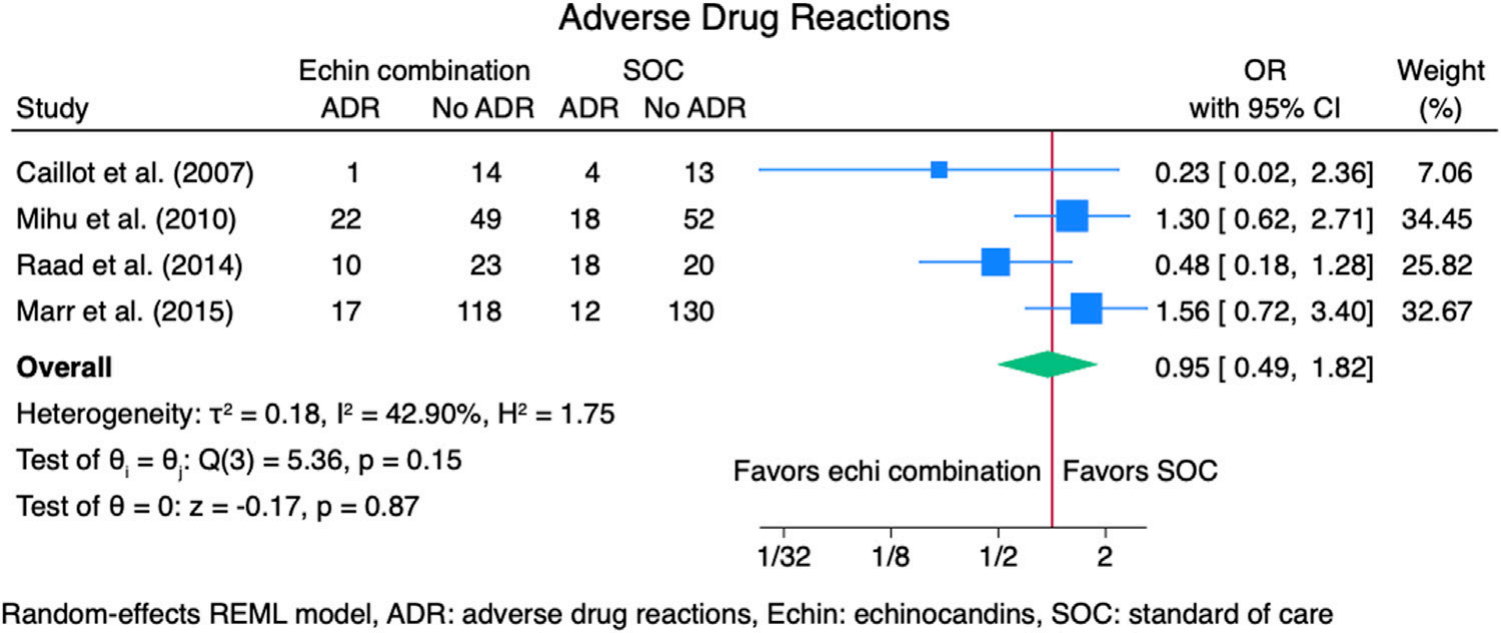
Meta-analysis of adverse drug reactions.

Four studies (Caillot et al., 2007; Marr et al., 2015; Mihu et al., 2010; Raad et al., 2015) reported outcomes for adverse drug reactions (ADRs), including 521 patients: 254 in the echinocandins combination group and 267 in the SOC monotherapy group. The pooled OR for ADRs was not statistically different for the echinocandins combination group compared to SOC monotherapy: OR 0.95 (95% CI: 0.49–1.82, *p* = 0.87, Figure 8). Reported ADRs include hepatotoxicity and nephrotoxicity that was not statistically different between groups (Raad et al., 2015). March 2015 found more patients in the combination group had hepatobiliary ADRs (12.7% vs. 8.4%) (Marr et al., 2015). Mihu et al. found numerically higher ADRs in the echinocandins combination group compared to SOC monotherapy (31% vs. 26%, *p* = 0.08) (Mihu et al., 2010). Abnormalities in bilirubin, alkaline phosphatase, and liver enzymes were not statistically different for the echinocandins combination group compared to SOC monotherapy. However, Mihu et al. found more patients with creatinine elevation in the echinocandin combination group than in SOC monotherapy (*p* = 0.01). Caillot et al. found fewer ADRs in the echinocandins combination group compared to SOC monotherapy. Adverse drug reactions include elevation in serum creatinine level, hypokalemia, and infusion-related reactions (Caillot et al., 2007).

### 3.4 Publication bias

For the clinical cure, the funnel plot of standard error against the effect estimate demonstrates symmetry in general, Supplementary Figure S5. The Egger test also revealed no statistically significant publication bias (*p* = 0.16). When assessing publication bias for mortality rate, the funnel plot of the standard error against the effect estimate generally demonstrates symmetry (Supplementary Figure S6), supported by the Egger test that revealed no statistically significant publication bias (*p* = 0.78).

## 4 Discussion

Invasive aspergillosis infections are challenging to treat and are associated with significant morbidity and mortality (Denning, 2024). Some studies have demonstrated that combining an echinocandin antifungal agent with the SOC improves treatment outcomes of IAI; however, the evidence is inconclusive, and there is a gap in the literature (Singh et al., 2006; Zhang et al., 2014). This study evaluated the effectiveness and safety of echinocandins combination therapy compared to SOC monotherapy for the treatment of IAI quantitatively by meta-analysis. We found that the rate of clinical cure and mortality were not statistically different for echinocandins combination therapy compared to SOC monotherapy for treating IAI. Although not statistically significant, there was a trend for a better clinical cure rate with echinocandins in combination with the SOC.

Combination antifungal therapy with echinocandins and SOC is appealing for treating IAI because it is difficult to treat (Patterson et al., 2016). Although we found no difference in clinical cure rate in our meta-analysis, there is a signal for better outcomes for echinocandins with SOC combination (OR> 1). An RCT by Caillot et al. (2007) evaluated the combination therapy of caspofungin with amphotericin in hematologic patients with IAI and revealed that the combination therapy had statistically significantly more favorable clinical cure compared to SOC monotherapy; the clinical cure rates were 67% and 27% (*p* = 0.028) for combination therapy and SOC monotherapy, respectively. The authors also found that the survival rate was 100% for combination therapy and 80% for SOC monotherapy. A study by Marr et al. (2004) evaluated combination therapy for treating IAI infection and found that combination therapy with echinocandins and triazole antifungals was associated with improved survival compared to SOC monotherapy; relative risk (RR) 0.42 (95% CI, 0.17–1.1, *p* = 0.048). A study by Singh et al. (2006) found a numerically higher survival rate for patients treated with combination therapy of caspofungin and voriconazole; however, the difference was not statistically significant; 67.5% for combination therapy and 51% for SOC monotherapy, *p* = 0.11. To elaborate, a study by Kontoyiannis et al. (2003) evaluated the efficacy and safety of combination therapy of caspofungin with amphotericin in patients with hematologic malignancies and found that the overall clinical cure rate was 42%, and the mortality rate was zero. The low clinical cure rate was attributed to a small sample size (42 patients). Notably, a randomized pragmatic superiority trial (IA-DUE) evaluated the azole-echinocandins combination for treating IAI was completed on 1 May 2024 (ClinicalTrials.gov). The trial aimed to assess azole-echinocandin combination therapy for IAI, and patients were randomized to receive either azole antifungal + anidulafungin or azole antifungal monotherapy. Of note is that the study’s findings have not been published yet.

Our study expands the findings of a meta-analysis by Zhang et al. (2014), which evaluated the effectiveness of combination therapy for treating IAI in animal studies. The authors found that echinocandins and triazole combination compared to echinocandins monotherapy prolonged survival for pooled animal studies, RR = 2.26 (95% CI, 1.79–2.87, *p* < 0.00001). That can be explained by the inadequate efficacy of echinocandin monotherapy for treating IAI, which is against the IDSA guideline recommendations (Patterson et al., 2016). On the contrary, when the authors compared the combination of echinocandins plus triazole with triazole monotherapy, they found no difference in survival, RR 1.19 (95% CI: 0.98–1.44, *p* = 0.08). Findings from the meta-analysis by Zhang et al. were translated into our study in humans since we did not find statistically significant differences in mortality for echinocandins combination therapy with SOC compared to SOC monotherapy, OR 0.90 (95% CI: 0.50–1.63, *p* = 0.73, Figure). The limitation in the meta-analysis by Zhang et al. that was overcome in our study is the lack of meta-analysis for human studies. Furthermore, another meta-analysis by Panackal et al. (2014) evaluated the salvage combination of all antifungal therapies for IAI (that include triazole with amphotericin combination and found that the combination therapy for IAI improved 12-weeks survival: Peto OR 1.80 (95% CI: 1.08–3.01). Their findings contradict our findings since we found no difference in mortality for echinocandin combination therapy compared to SOC monotherapy. Limitations of this meta-analysis are that it used all combination therapies together; thus, estimates for studies evaluating echinocandins with SOC combination therapies are not available, and they used a fixed-effect model in their meta-analysis, which cannot adjust for heterogeneity across studies. Lastly, a network meta-analysis by Liu et al. (2024) found that combination therapy of amphotericin with caspofungin was associated with the best probability of favorable response: the surface under the cumulative ranking curve was 84.1%; mean rank, 2.6. However, the limitation of this study was it performed an indirect (artificial) comparison.

Several studies evaluated risk factors for mortality due to IAI. Raad et al. (2015) found intensive care unit admission for primary and salvage therapy was a risk factor for mortality for IAI. Marr et al. (2015) found that elevated serum galactomannan predicted mortality related to IAI. On the contrary, (Raad et al., 2008) found posaconazole therapy was associated with favorable clinical response. Moreover, Singh et al. (2006) found that renal failure was associated with mortality.

Our findings from pooled studies revealed that the echinocandins combination group had a similar safety profile to SOC monotherapy (OR 0.95, 95% CI: 0.49–1.82, *p* = 0.87). Our findings contradict toxicity results reported by Raad et al. (2008) since they found that caspofungin combination with amphotericin was associated with significantly higher rates of hepatotoxicity and renal injury compared to posaconazole, *p* ≤ 0.02. The findings by Raad et al. can be explained by the presence of amphotericin in the combination regimen, which is highly associated with hepatoxicity and nephrotoxicity. The combination therapy with echinocandins is unlikely to increase the incidence of ADRs.

Our meta-analysis is the first study to evaluate the effectiveness and safety of combination antifungal drugs for treating IAI in humans, precisely the combination of echinocandins with SOC. We performed subgroup meta-analyses to assess the outcomes of our study further. Our meta-analysis has limitations. We included all studies, including observational studies; however, all were rated high quality. The population in our meta-analysis who acquired IAI was heterogeneous, including patients with hematologic malignancies and transplant patients. Different formulations for amphotericin were used in the included studies, and they could have distinct safety profiles. Furthermore, the criteria for diagnosing IAI used in the included studies may differ slightly, affecting the homogeneity of patients with IAI. However, all heterogeneities in our meta-analysis were adjusted for our meta-analysis’s heterogeneity by using the random-effects model. We could not evaluate outcomes based on the IAI site. Lastly, we included only studies published in the English language.

## 5 Conclusion

Treatment of IAI is challenging and requires new therapeutic approaches to prevent treatment failure and complications. Using combination therapy of echinocandins antifungal agents with the SOC has been an appealing option and recommended in the IDSA IAI guideline. Our meta-analysis found no differences in clinical cure and mortality rate when using combination therapy of echinocandin antifungal agents with the SOC compared to SOC monotherapy. However, regarding clinical cure rate, we found a signal for better outcomes with combination therapy of echinocandin antifungal agent with SOC. We also found that combination therapy with echinocandins and SOC did not result in statistically significantly more ADRs compared to SOC monotherapy. Future studies should evaluate the cost-effectiveness of combination therapy with echinocandins antifungal agents.

## Data Availability

The original contributions presented in the study are included in the article/Supplementary Material, further inquiries can be directed to the corresponding author.
